# Red and processed meat and pancreatic cancer risk: a meta-analysis

**DOI:** 10.3389/fnut.2023.1249407

**Published:** 2023-09-27

**Authors:** Yudi Sun, Xinyi He, Yan Sun

**Affiliations:** Department of Gastroenterology, Shengjing Hospital of China Medical University, Shenyang, Liaoning, China

**Keywords:** red meat, processed meat, pancreatic cancer, daily diet, meta-analysis

## Abstract

**Background:**

The relationship between red and processed meat consumption and pancreatic cancer risk is controversial and no study has looked specifically at the correlation for 6 years. We conducted a meta-analysis to summarize the evidence about the association between them.

**Methods:**

We systematically searched PubMed, Embase and Cochrane Library for studies of red or processed meat consumption and pancreatic cancer published from December 2016 to July 2022. We performed random-effects models to pool the relative risks from individual studies. Subgroup analyses were used to figure out heterogeneity. We also performed publication bias analysis.

**Results:**

Seven cohort studies and one case–control study that contained a total of 7,158 pancreatic cancer cases from 805,177 participants were eligible for inclusion. The combined RRs (95% CI) comparing highest and lowest categories were 1.07 (95% CI: 0.91–1.26; *p* = 0.064) for red meat and 1.04 (95% CI: 0.81–1.33; *p* = 0.006) for processed meat with statistically significant heterogeneity.

**Conclusion:**

This meta-analysis suggested that red and processed meat consumption has no relationship with pancreatic cancer risk.

## Introduction

Pancreatic cancer, ranking as the seventh leading cause of mortality from malignancies globally owing to its unfavorable prognosis, exhibits a higher incidence rate in nations with a High Human Development Index (HDI) countries (1). This trend can be attributed to the escalating prevalence of obesity, diabetes, and alcohol consumption within these high HDI countries alongside advancements in diagnostic techniques and enhanced cancer registration protocols (2). According to an analysis encompassing 28 European nations, pancreatic carcinoma is projected to surpass breast malignancy and become the third most prevalent fatal neoplasm by 2025 (3). Therefore, it is imperative to identify risk factors for pancreatic cancer, making it of paramount importance.

Strong evidence suggests that consumption of red and processed meat may elevate the risk of pancreatic cancer due to its high content of heterocyclic amines (HCAs), polycyclic aromatic hydrocarbons (PHAs), and N-nitroso compounds (NOCs). In April 2017, Zhao et al. conducted a systematic review and meta-analysis investigating the association between intake of red and processed meat and the risk of pancreatic cancer. The analysis included a total of 28 cohort and case–control studies published before February 2016. Among the case–control studies, higher consumption of red meat and processed meat exhibited a positive correlation with pancreatic cancer risk (Red meat, RR: 1.38, 95% CI: 1.05–1.81; Processed meat, RR: 1.62, 95% CI: 1.17–2.26); however, no overall association was observed in cohort studies (Red meat, RR: 1.12, 95% CI: 0.98–1.28; Processed meat, RR: 1.09, 95% CI: 0.96–1.23). Dose–response analysis indicated that every additional daily intake increment by 100 g in red meat was associated with an 11% increased risk for developing pancreatic cancer while each 50 g/d increase in processed meat intake led to an 8% rise in this risk. The findings from this study align with dietary guidelines while providing more comprehensive evidence based on robust epidemiological data. However, no clear relationship has been established between recent six-year trends in red or processed meat consumption and pancreatic cancer risk.

Therefore, we performed an updated meta-analysis incorporating epidemiological studies published from February 2016 to July 2022 (including cohort and case–control studies) to investigate associations between red or processed meat consumption and incidence or mortality of pancreatic cancer. Additionally, the potential influence of factors such as sex (men vs. women), geographic area (USA vs. Italy), duration of follow-up (less than 20 years vs. more than 20 years) and adjustments for alcohol intake, smoking, BMI, diabetes, family history pancreatic cancer, energy intake and physical activity were investigated.

## Methods

### Search strategy

Articles published from December 2016 to October 2022 were systematically searched in PubMed, Embase and Cochrane Library. The search strategy was a combination of medical subject headings and free text words. Following medical subject terms were used: “Pancreatic Neoplasms,” “Red Meat” and “Meat Products.” Free text words including “red meat*,” “beef,” “pork,” “lamb,” “mutton,” “veal,” “pancreatic neoplasm*,” “Cancer of Pancreas,” “pancreas cancer*,” “pancreatic cancer*,” “Cancer of the Pancreas,” “diet*,” “food*” and “processed meat.” Moreover, we reviewed the reference lists from the included articles and those from previous meta-analysis to identify additional relevant studies.

### Study selection

Studies were included in our meta-analysis when they meet the following criteria:

1. Be a cohort or case–control study.

2. Provide the 95% confidence intervals and adjusted estimates of the relative risk (RR) (or any statistical indicator to compute them like hazard ratio, odds ratio or risk ratio) for red and/or processed meat of pancreatic cancer incidence or mortality.

3. The study was published from December 2016 to July 2022.

4. The study was published in English. We considered “ham,” “sausage,” “bacon,” “salami” and “hot dogs” as equivalent to “processed meat.” When there were multiple published reports from the same study population, we chose the one with the largest population. For studies that researched for more than 1 cohort, the data of each cohort were selected (Supplementary Table S1) (4).

### Data extraction and quality assessment

Two investigators independently extracted the following data from each study: the first author’s name, the year of publication, the country where the study was performed, age of subjects, number of participants, follow-up time, type of the meat and their consumption strategy, adjusted RR/OR/HR with the corresponding 95% CI (highest to lowest) and other influencing factors. When some studies gave the RR values of processed red meat and unprocessed red meat, we extracted unprocessed red meat as the data of red meat in that study. When a study provided RR values for all age groups vs. people aged 50 years, we extracted data for all age groups (Supplementary Table S1) (5).

The Newcastle-Ottawa Scale (NOS) was applied to assess the study quality (6), which ranged from 1 to 9 stars. We considered studies with NOS scores higher than 7 to be of high methodologic quality.

### Statistical analysis

Due to variations in the reporting methods of effect size and 95% CI across studies, we estimated these values by comparing the highest with the lowest consumption categories (e.g., quartile or quintile). For simplicity, we use the term RR (relative risk) for all estimates in this manuscript since hazard ratios in some cohort studies and odds ratios in case–control studies are closely approximate relative risks (7, 8) when pancreatic cancer incidence or mortality is low. The corresponding RR for each study was assigned to the highest level of red and processed meat intake within each category. In cases where the highest category was open-ended, we considered it equivalent to the adjacent interval. We employed a random effects model to calculate RRs and 95% CI. To stabilize variance and standardize distribution, RRs and their corresponding standard errors were converted into natural logarithm and then the logarithm is removed again to be reduced. To obtain a single RR from each study, RRs were combined when separate relative risk estimates were provided for populations that were over 60 or up to 60 years old (Supplementary Table S1; 9). The outcomes are presented as a forest plot with 95% CIs.

Q and I^2^ statistics were used to evaluate statistical heterogeneity among studies (10), when *p* < 0.10 and I^2^ > 50%, the heterogeneity considered to be statistically significant. To investigate other influencing factors for the association between red or processed meat consumption and pancreatic cancer, we conducted subgroup analyses by sex (men vs. women), geographic area (USA vs. Italy), duration of follow-up (<20 years vs. >20 years) and adjustments for alcohol intake, smoking, BMI, diabetes, family history pancreatic cancer, energy intake and physical activity.

To evaluate the stability of the results, we performed sensitivity analyses of statistically significant results, which were more stable if the pooled effect size changed little and none of the 95% confidence intervals crossed 1 after removing a single study.

We also used Egger’s regression asymmetry test (11) and Begg-Mazumdar test (12) to assess publication bias, *p* < 0.05 considered statistically significant.

All statistical analyses were performed with STATA version 17.0 software (Stata Corporation, College Station, TX).

## Results

**Figure 1:**
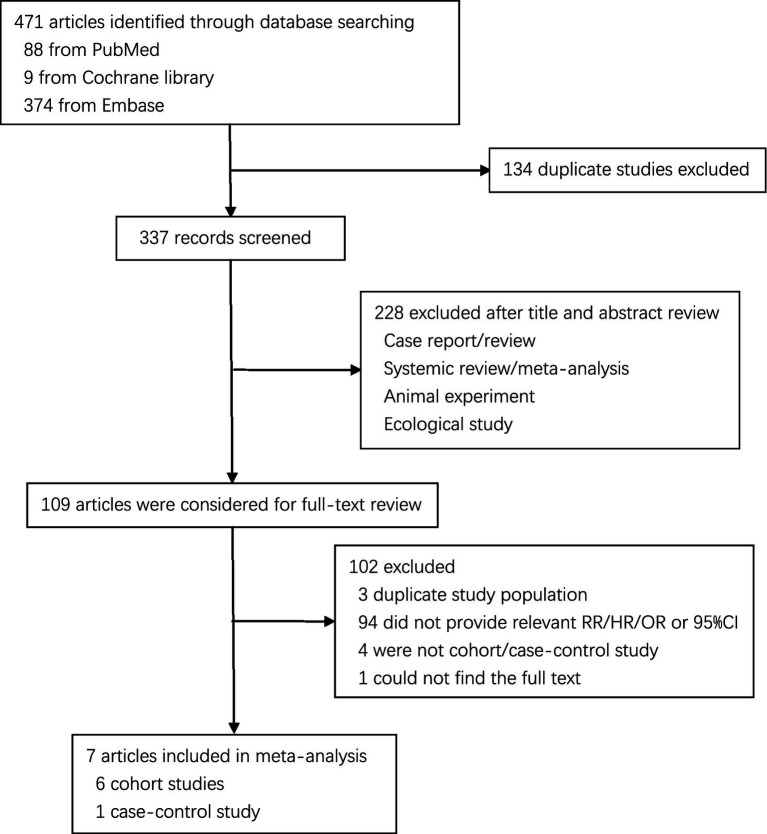
Flow chart of study selection.

The literature search generated 471 records, of which 109 were considered potentially valuable (Figure 1). Finally, a total of 7 articles were included in the meta-analysis (6 cohorts and 2 case–controls) and reported 12 separate data (7 red meat and 5 processed meat) on the association between red or processed meat with pancreatic cancer (4, 5, 9, 13–17).

### Study characteristics

The characteristics of included studies are outlined in Supplementary Table S1. We included 8 studies that contained a total of 7,158 pancreatic cancer cases from 805,177 participants. In eight included studies, all of them were carried in HDI countries, including six in USA and two in Italy. Seven of them consisted of men and women, of only women in one study. There are four reported the relationship between two types of meat with pancreatic cancer risk in addition to three only reported the red meat and one only reported the processed meat.

### Red meat

#### Highest vs. lowest intake category

**Figure 2:**
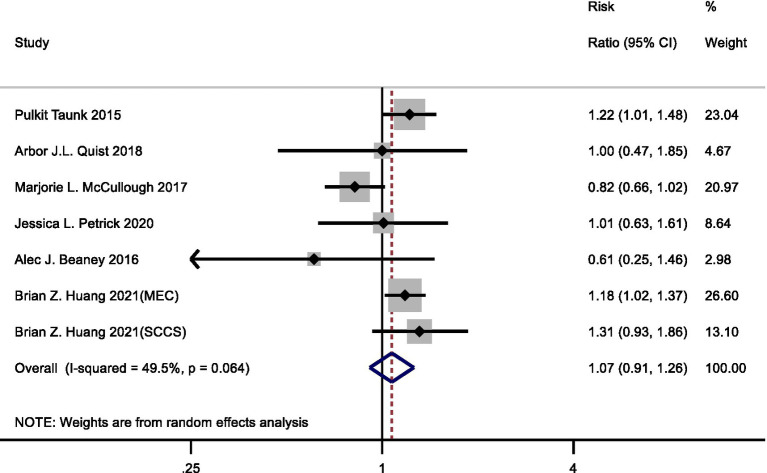
Forest plot of red meat consumption (highest versus lowest category) and pancreatic cancer risk.

Seven cohort studies examined the relationship between red meat consumption with pancreatic cancer risk. In the meta-analysis comparing highest vs. lowest intake category, the summary RR for pancreatic cancer risk was 1.07 (95%CI: 0.91–1.26) with statistically significant heterogeneity observed (*I*^2^ = 49.5%, *p* = 0.064) (Figure 2).

#### Subgroup analysis

Subgroup analyses are shown in Table 1. In the subgroup analysis by sex, the RRs were 1.10 (95% CI: 0.71–1.70) in men and 0.92 (95% CI: 0.74–1.15) in women. In the subgroup analysis by geographic area, the RRs were 1.11 (95% CI: 0.93–1.33) for the studies in the USA and 0.61 (95% CI: 0.25–1.46) for the studies in Italy. In the subgroup analysis by duration of follow-up, the RRs were 1.18 (95% CI: 1.03–1.34) for those with more than 20 years of follow-up. Moreover, RRs were 1.19 (95% CI: 1.07–1.33) and 1.20 (95% CI: 1.05–1.37) for the studies adjusted for alcohol intake and family history pancreatic cancer, respectively. In other subgroup analyses, heterogeneity could be observed, but in adjustments for physical activity, the finding remained robust.

**Table 1:** Summary relative ratio (RR) and 95% confidence intervals (95% CI) of highest vs. lowest category of red and processed meat consumption and pancreatic cancer risk by subgroups.

Subgroups		Red meat						Processed meat					
		n	RR (95%CI)	I2% (within)	P1	I2% (between)	P2	n	RR (95%CI)	I2% (within)	P1	I2% (between)	P2
All studies		7	1.07 (0.91–1.26)	49.5	0.064	–	–	5	1.04 (0.81–1.33)	72	0.006	–	–
Sex						62.7	0.045					0	0.443
	Men	2	1.10 (0.71–1.70)	80.9	0.022			2	0.92 (0.69–1.23)	56.8	0.128		
	Women	2	0.92 (0.74–1.15)	0	0.41			2	0.92 (0.74–1.14)	0	0.557		
Geographic area						56.3	0.182					72	0.006
	USA	6	1.11 (0.93–1.33)	58.1	0.036			3	0.91 (0.77–1.07)	23.8	0.269		
	Italy	1	0.61 (0.25–1.46)	–	–			2	1.45 (1.16–1.82)	0	0.873		
Duration of follow-up						44.6	0.094					72.5	0.006
	<20 years	3	0.95 (0.68–1.32)	74.6	0.02			3	0.95 (0.77–1.18)	44.3	0.166		
	>20 years	4	1.18 (1.03–1.34)	0	0.803			2	1.11 (0.61–2.02)	81	0.022		
Adjustment for alcohol intake						45.4	0.089					9.1	0.354
	Yes	4	1.19 (1.07–1.33)	0	0.841			3	1.00 (0.85–1.17)	0	0.568		
	No	3	0.85 (0.69–1.03)	0	0.535			2	0.97 (0.59–1.58)	51.2	0.152		
Adjustment for smoking						–	–					–	–
	Yes	7	1.08 (0.93–1.25)	44.6	0.094			5	0.95 (0.82–1.10)	16	0.313		
	No	0	–	–	–			0	–	–	–		
Adjustment for BMI						45.4	0.089					72.4	0.006
	Yes	6	1.11 (0.97–1.27)	36.7	0.162			4	1.01 (0.77–1.33)	78.2	0.003		
	No	1	0.59 (0.29–1.19)	–	–			1	1.42 (0.69–2.91)	–	–		
Adjustment for diabetes						–	–					72.5	0.006
	Yes	7	1.08 (0.93–1.25)	44.6	0.094			4	0.93 (0.78–1.11)	26.9	0.251		
	No	0	–	–	–			1	1.46 (1.15–1.85)				
Adjustment for family history pancreatic cancer						45.4	0.089					–	–
	Yes	2	1.20 (1.05–1.37)	0	0.587			5	1.04 (0.81–1.34)	72.4	0.006		
	No	5	0.98 (0.78–1.23)	50.1	0.091			0	–	–	–		
Adjustment for energy intake						44.6	0.094					72.5	0.006
	Yes	3	1.02 (0.73–1.43)	48.9	0.141			4	1.14 (0.86–1.50)	63.7	0.041		
	No	4	1.08 (0.88–1.32)	55.5	0.081			1	0.82 (0.66–1.02)	–	–		
Adjustment for physical activity						42.1	0.11					73.3	0.005
	Yes	4	0.87 (0.73–1.05)	0	0.709			2	1.05 (0.54–2.03)	58	0.123		
	No	3	1.21 (1.08–1.35)	0	0.854			3	1.06 (0.78–1.45)	83.9	0.002		

#### Sensitivity analysis and publication bias

**Figure 3:**
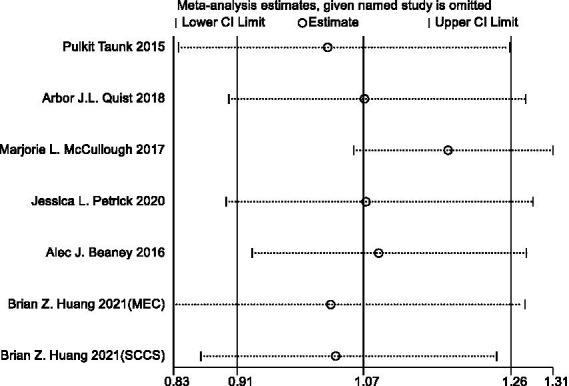
Sensitivity analyses of red meat consumption and pancreatic cancer risk.

**Figure 4:**
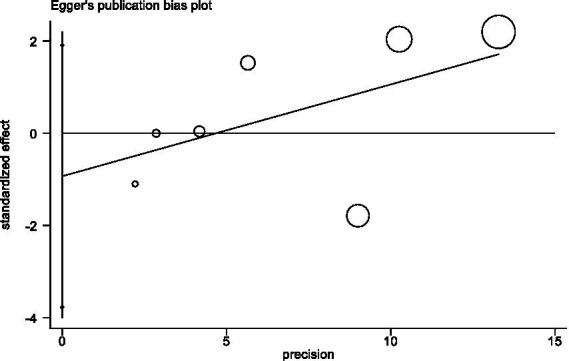
Egger’s test evaluating publication bias of red meat consumption and pancreatic risk.

In sensitivity analyses, the association between red meat intake and pancreatic cancer risk was not significantly altered after removing each single study (Figure 3). The Egger’s test (*p* = 0.438) and Begg’s test (*p* = 0.230) did not detect publication bias (Figure 4).

### Processed meat

#### Highest vs. lowest intake category

**Figure 5:**
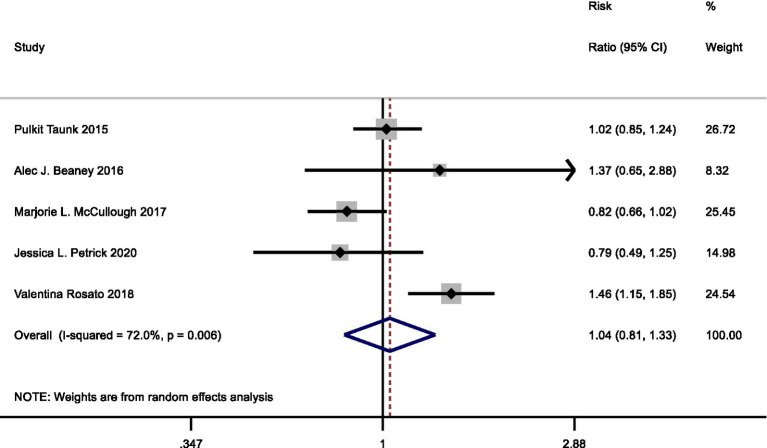
Forest plot of processed meat consumption (highest vs. lowest category) and pancreatic cancer risk.

Four cohort studies and one case–control study examined the relationship between processed meat consumption with pancreatic cancer risk. In the meta-analysis comparing highest vs. lowest intake category, the summary RR for pancreatic cancer risk was 1.04 (95%CI: 0.81–1.33) with statistically significant heterogeneity observed (*I*^2^ = 72.0%, *p* = 0.006) (Figure 5).

#### Subgroup analysis

Subgroup analyses are shown in Table 1. In the subgroup analysis by sex, the RRs were 0.92 (95% CI: 0.69–1.23) in men and 0.92 (95% CI: 0.74–1.14) in women. In the subgroup analysis by geographic area, the RRs were 0.91 (95% CI: 0.77–1.07) for the studies in the USA and 1.45 (95% CI: 1.16–1.82) for the studies in Italy. In other subgroup analyses, heterogeneity could be observed, but in adjustments for alcohol intake, the finding remained robust.

#### Sensitivity analysis and publication bias

**Figure 6:**
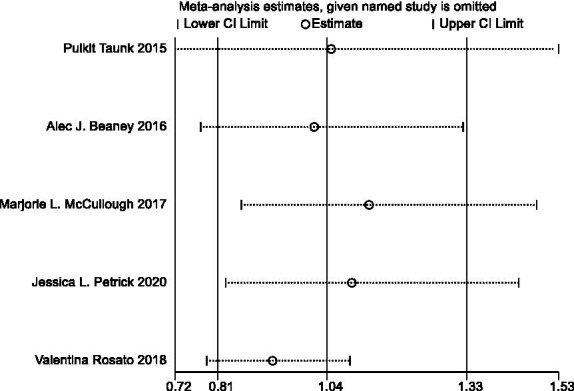
Sensitivity analyses of processed meat consumption and pancreatic cancer risk (all studies).

**Figure 7:**
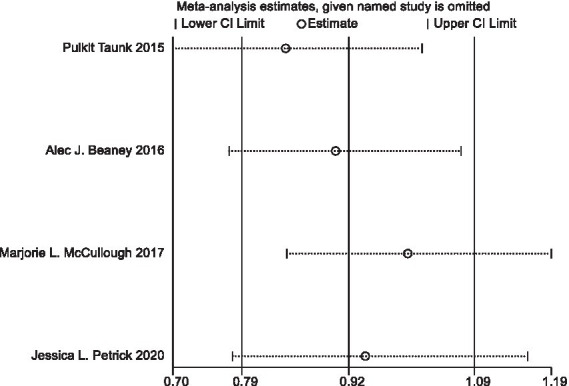
Sensitivity analyses of processed meat consumption and pancreatic cancer risk (all cohort studies).

**Figure 8:**
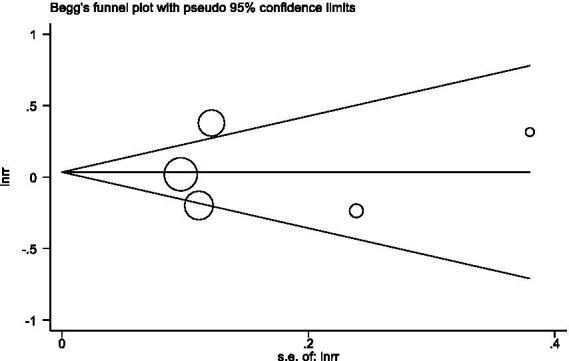
Begg’s test evaluating publication bias of processed meat consumption and pancreatic risk.

In sensitivity analyses, the association between processed meat intake and pancreatic cancer risk was not significantly altered after removing each single study (Figure 6). After deleting the only one case–control study (Supplementary Table S1) (16), the results remained stable (Figure 7). The Egger’s test (*p* = 0.932) and Begg’s test (*p* = 0.806) did not detect publication bias (Figure 8).

## Discussion

In our meta-analysis, we did not find any significant association between the consumption of red meat or processed meat and the risk of pancreatic cancer. However, subgroup analyses based on a limited number of studies indicated a positive association between processed meat consumption and pancreatic cancer incidence in Italy, while no such relationship was observed in the USA. Additionally, alcohol intake or family history of pancreatic cancer has been identified as independent factors associated with an increased incidence of pancreatic cancer; therefore, we adjusted our analysis to explore their influence. Surprisingly, our results revealed a positive correlation between red meat consumption and alcohol intake or family history of pancreatic cancer, but no such relationship was found for processed meat. The subgroup analyses conducted in this study were unable to fully account for potential sources of interstudy heterogeneity, as statistically significant differences in histological subtype, number of samples, or study quality were not observed. In the sensitivity analysis of the literature on the relationship between pancreatic cancer and processed meat, we included one case control study due to its limited number (Supplementary Table S1) (16). After re-performing the sensitivity analysis on the remaining cohort studies, our results remained consistent and robust.

Our findings are consistent with those of numerous other meta-analyses that have reached similar conclusions regarding the association between red and processed meat consumption and pancreatic cancer risk. For instance, Farvid et al. performed a systematic review and meta-analysis of 148 prospective studies published up to December 2020 to summarize the evidence on the relationship between consumption of red meat (unprocessed), processed meat, total red meat, processed meat, and the incidence of various cancers. By utilizing random-effects models to summarize relative risks for the highest and lowest intake categories for each exposure variable, they concluded that neither intake of red meat alone (unprocessed) nor processed meat alone was associated with an increased risk of pancreatic cancer (18). Han et al. in their dose–response meta-analysis comprising 118 cohort studies reporting associations between consumption of unprocessed red meat and processed meat and mortality/incidence rates for six different cancers including pancreatic cancer since April 2019, calculated pooled relative risks (RR) along with corresponding 95% confidence intervals (CI). Their analysis demonstrated that reducing weekly intake by three servings per week had only a minimal absolute effect on cancer mortality or incidence related to both red meat and processed meat consumption (19). Zeraatkar et al., who included 14 randomized trials comparing lower vs. higher intakes of either red or processed meat among adults in terms of cardiovascular disease and cancer incidence rates found that one randomized trial indicated a low red meat diet may have limited impact on pancreatic cancer risk specifically among women (20). Additionally, Vernooij et al. found no statistically significant risk of pancreatic cancer incidence for dietary patterns with lower intake of red meat and processed meat (21).

However, most studies suggest that there is a biologically plausible positive association between the consumption of red and processed meat and pancreatic cancer. Several mechanisms have been proposed to explain the potential role played by red and processed meat in increasing pancreatic cancer risk. For instance, it is well-established that red meat contains heme iron, which stimulates the production of N-nitroso compounds (NOCs) by bacteria in the large intestine (22). Moreover, red meat consumption promotes DNA adduct formation, leading to epigenetic changes in DNA. On the other hand, processed meat contains higher levels of nitrite/nitrate and sodium compared to unprocessed meat (23), which further enhances NOCs production. Additionally, cooking methods such as high-temperature frying, grilling or smoking result in the formation of heterocyclic amines (HAAs) and polycyclic aromatic hydrocarbons (PAHs) (24). Animal studies have demonstrated that these compounds may induce DNA adduct formation and interfere with apoptosis processes, potentially promoting carcinogenesis. Furthermore, both red and/or processed meats are rich sources of saturated fat; animal experiments have shown that rats fed a high-fat diet develop more pancreatic cancer compared to those on a low-unsaturated fat diet (25, 26). Observational studies also support a positive association between animal fat intake and pancreatic cancer incidence (27). This could be attributed to excess saturated fat inducing alterations in gut microbiota composition, thereby activating proinflammatory pathways and leading to inflammation, which is known as risk factors for cancer (28). Additionally, prolonged cooking at high temperatures enhances the formation of N- (carboxymethyl) lysine (CML) advanced glycation end products (CML AGEs) in food (29). CML AGEs may lead to insulin resistance, oxidative stress, and chronic inflammation (30–32). It is hypothesized that CML AGEs may contribute to the development of pancreatic cancer by altering the interstitial environment of tissues (33). Moreover, several persistent organic pollutants (POPs) are commonly detected in meat, which are carcinogenic. Studies suggest that consuming lamb or other POPs may increase the risk of cancer (34). Lastly, red meat contains binding forms of Nonhuman sialic acid N-glycolylneuraminic acid (Neu5Gc) and methionine that can be incorporated into human tissues through metabolism, leading to inflammation (35, 36). This mechanism could potentially explain why consumption of red meat is associated with an increased risk of pancreatic cancer.

Our study possesses several strengths. Firstly, our search strategy was meticulously detailed. Secondly, it was conducted by two independent reviewers for data selection and extraction, with consultation from a third investigator in case of disagreements after discussion, thereby minimizing bias and error. Thirdly, we have a substantial sample size that allows for more robust conclusions regarding the association between intake of red and processed meat and pancreatic cancer risk. Fourthly, the studies included in this article were predominantly cohort studies (with only one being a case–control study), which reduces the potential for recall and selection biases. Lastly, subgroup analyses were performed to investigate the sources of heterogeneity.

However, our study also has certain limitations. Firstly, the original studies encompassed both cohort studies and case–control studies; therefore recall bias, interviewer bias, and inaccurate measures of dietary consumption in case–control studies may impact the outcomes concerning processed meat consumption and pancreatic cancer risk. Secondly, there might be selection bias within the study population as it primarily focused on two high human development index (HDI) countries, the United States and Italy, where a higher incidence of pancreatic cancer is associated with a higher quality of life. Additionally, individuals in these countries tend to consume more red and processed meat compared to those in developing nations which could potentially inflate associations observed. Moreover, some participants were recruited from health care registries who generally exhibit greater attention toward healthy living practices thus reducing correlations between variables studied here. Thirdly, due to variations among original studies regarding specific daily meat consumption details, the inclusion criteria uniformly extracted highest and lowest levels of relative risk (RR) intake. Fourthly, certain studies accounted for potential confounding factors, including gender, alcohol consumption, smoking habits, and body mass index (BMI), while others did not. Fifthly, it is worth noting that our findings may have been influenced by imprecise measurements of meat intake in the original studies or variations in meat cooking methods, which could have impacted the overall relative risk estimation. Furthermore, since the original studies included in our study employed different unit categories (e.g., portion size and time), standardization was not feasible for extracting average values for red meat or processed meat from each stud; thus preventing us from conducting a dose–response analysis. We hope that future studies can provide more detailed investigations into the dose–response relationship between red/processed meat consumption and pancreatic cancer.

## Conclusion

Our meta-analysis of cohort and case–control studies revealed no significant association between red meat and processed meat consumption and pancreatic cancer risk. However, considering the dietary guidelines proposed by the NutriRECS consortium in 2019 (37) and conclusions drawn by other researchers, further investigations are warranted to validate this relationship.

## Data availability statement

The original contributions presented in the study are included in the article/Supplementary material, further inquiries can be directed to the corresponding author.

## Author contributions

YaS and YuS designed the study. YuS contributed to the literature database search, data collection, data extraction, data analysis, and writing of the manuscript. XH performed the data extraction and check of the results. All authors contributed to the article and approved the submitted version.
