# Supplementary material for: Red and processed meat and pancreatic cancer risk: a meta-analysis
Source: Front Nutr. 2023 Sep 27;10:1249407. doi: 10.3389/fnut.2023.1249407 (PMC10565855; doi:10.3389/fnut.2023.1249407)
Supplement: Supplementary file 1 [file Table_1.DOCX]

| **Supplementary Table Characteristics of the studies included in the meta-analysis** | | | | | | | |
| --- | --- | --- | --- | --- | --- | --- | --- |
| **First author, publication year, country** | **Study type** | **No. of case patients (outcome)** | **Cohort size and duration of follow-up** | **Type of meat and exposure categories** | **Adjust RR/OR/HR (95%CI)** | **Controlled variables** | **Quality score** |
| Pulkit Taunk^1^,  2015,  United States | Cohort study | 1417 (895 males, 522 females)  (incidence) | 322846 men and women; 11y | Red and processed meat;  Quintile  (g/1,000 kcal) |  | Age, sex, energy, smoking, BMI, education, race, self-reported diabetes, alcohol use and energy-adjusted saturated fat | 9 |
|  |  |  |  | Q1  Q2  Q3  Q4  Q5 | **Red meat**  **(combined)**  1.00 (ref.)  1.06 (0.88-1.26)  1.11 (0.92-1.32)  1.18 (0.99-1.42)  1.22 (1.01-1.48) |  |  |
|  |  |  |  | Q1  Q2  Q3  Q4  Q5 | **(men)**  1.00 (ref.)  1.02 (0.81-1.28)  1.14 (0.91-1.44)  1.20 (0.95-1.51)  1.36 (1.07-1.73) |  |  |
|  |  |  |  | Q1  Q2  Q3  Q4  Q5 | **(women)**  1.00 (ref.)  1.11 (0.84-1.47)  1.05 (0.78-1.40)  1.17 (0.87-1.56)  1.01 (0.74-1.38) |  |  |
|  |  |  |  | Q1  Q2  Q3  Q4  Q5 | **Processed**  **(combined)**  1.00 (ref.)  1.29 (1.08-1.53)  1.23 (1.02-1.46)  1.24 (1.04-1.49)  1.02 (0.85-1.24) |  |  |
|  |  |  |  | Q1  Q2  Q3  Q4  Q5 | **(men)**  1.00 (ref.)  1.33 (1.07-1.66)  1.17 (0.93-1.47)  1.26 (1.00-1.58)  1.05 (0.83-1.33) |  |  |
|  |  |  |  | Q1  Q2  Q3  Q4  Q5 | **(women)**  1.00 (ref.)  1.22 (0.91-1.62)  1.33 (1.00-1.76)  1.23 (0.91-1.65)  0.98 (0.72-1.33) |  |  |
| Alec J. Beaney^2^,  2016,  Italy | Cohort study | 1532 (incidence) | 23133 men and women; 17y | Red and processed meat;  Quintiles (g/d) and age |  | Sex, age, diabetes mellitus, smoking, energy, BMI, physical activity, dietary antioxidant intake, cooking methods, plasma | 7 |
|  |  |  |  | Q1: 0 to <8.6  Q2: 8.6 to <23.1  Q3: 23.1 to <36.8  Q4: 36.8 to <55.7  Q5: 55.7-349.3 | **Red**  **(Age<60)**  1.00 (ref)  3.23 (0.65-16.12)  2.13 (0.39-11.73)  4.32 (0.91-20.59)  4.62 (0.96-22.30) |  |  |
|  |  |  |  | Q1: 0 to <8.6  Q2: 8.6 to <23.1  Q3: 23.1 to <36.8  Q4: 36.8 to <55.7  Q5: 55.7-349.3 | **(Age>=60)**  1.00 (ref)  0.51 (0.23-1.12)  0.38 (0.16-0.89)  0.80 (0.41-1.57)  0.24 (0.08-0.67) |  |  |
|  |  |  |  | Q1: 0 to <5.4  Q2: 5.4 to <14.2  Q3: 14.2 to <23.1  Q4: 23.1 to <36.1  Q5: 36.1-192.0 | **Processed**  **(Age<60)**  1.00 (ref)  1.56 (0.35-7.01)  2.64 (0.66-10.62)  3.35 (0.86-13.09)  3.73 (0.95-14.66) |  |  |
|  |  |  |  | Q1: 0 to <5.4  Q2: 5.4 to <14.2  Q3: 14.2 to <23.1  Q4: 23.1 to <36.1  Q5: 36.1-192.0 | **(Age>=60)**  1.00 (ref)  1.21 (0.55-2.68)  0.81 (0.34-1.92)  0.75 (0.31-1.83)  0.90 (0.37-2.17) |  |  |
| Arbor J.L. Quist^3^, 2018,  United States | Cohort study | 313 (incidence) | 34242 Iowa USA women; 25y | Red meat (g/d) |  | Age, race, PWS chlorinated, surface water, smoking, occupation, residence, BMI, education, diabetes, physical activity, oral contraceptive use, estrogen use, insulin use, diet | 7 |
|  |  |  |  | <45.47  45.47-79.10  79.11-120.17  120.18-198.56  >198.57 | 1.00 (ref)  0.75 (053-1.05)  1.03 (0.73-1.47)  1.00 (0.65-1.50)  1.00 (0.47-1.85) |  |  |
| Marjorie L. McCullough^4^,  2017,  United States | Cohort study | 1156 (incidence) | 138266 men and women; 15.7y | Red and processed meat;  Quintile (servings/week) |  | Age, race, education, BMI, diabetes, smoking, physical activity, dietary | 9 |
|  |  |  |  | Q1: ≤2.4/1.3  Q2: >2.4/1.3-4.2/2.6  Q3: >4.2/2.6-6.2/3.9  Q4: >6.2/3.9-9.1/5.9  Q5: >9.1/5.9 | **Red and processed meat**  **(combined)**  1.00 (ref)  0.90 (0.75-1.08)  0.84 (0.70-1.02)  0.89 (0.72-1.09)  0.81 (0.64-1.02) |  |  |
|  |  |  |  | Q1: ≤2.4/1.3  Q2: >2.4/1.3-4.2/2.6  Q3: >4.2/2.6-6.2/3.9  Q4: >6.2/3.9-9.1/5.9  Q5: >9.1/5.9 | **(men)**  1.00 (ref)  0.91 (0.71-1.17)  0.81 (0.61-1.06)  0.87 (0.65-1.15)  0.82 (0.58-1.14) |  |  |
|  |  |  |  | Q1: ≤2.4/1.3  Q2: >2.4/1.3-4.2/2.6  Q3: >4.2/2.6-6.2/3.9  Q4: >6.2/3.9-9.1/5.9  Q5: >9.1/5.9 | **(women)**  1.00 (ref)  0.88 (0.68-1.15)  0.89 (0.67-1.17)  0.91 (0.68-1.22)  0.80 (0.57-1.12) |  |  |
|  |  |  |  | Q1: ≤1.4/0.9  Q2: >1.4/0.9-2.3/1.6  Q3: >2.3/1.6-3.5/2.5  Q4: >3.5/2.5-5.2/3.8  Q5: >5.2/3.8 | **Red meat**  **(combined)**  1.00 (ref)  0.84 (0.70-1.00)  0.83 (0.69-1.00)  0.91 (0.75-1.10)  0.82 (0.66-1.02) |  |  |
|  |  |  |  | Q1: ≤1.4/0.9  Q2: >1.4/0.9-2.3/1.6  Q3: >2.3/1.6-3.5/2.5  Q4: >3.5/2.5-5.2/3.8  Q5: >5.2/3.8 | **(men)**  1.00 (ref)  0.77 (0.60-1.00)  0.87 (0.68-1.12)  0.93 (0.72-1.21)  0.87 (0.65-1.18) |  |  |
|  |  |  |  | Q1: ≤1.4/0.9  Q2: >1.4/0.9-2.3/1.6  Q3: >2.3/1.6-3.5/2.5  Q4: >3.5/2.5-5.2/3.8  Q5: >5.2/3.8 | **(women)**  1.00 (ref)  0.90 (0.69-1.16)  0.78 (0.59-1.03)  0.89 (0.67-1.17)  0.84 (0.62-1.15) |  |  |
|  |  |  |  | Q1: ≤0.5/0.1  Q2: >0.5/0.1-1.3/0.6  Q3: >1.3/0.6-2.4/1.2  Q4: >2.4/1.2-4.2/2.2  Q5: >4.2/2.2 | **Processed meat**  **(combined)**  1.00 (ref)  0.91 (0.76-1.09)  0.91 (0.76-1.10)  0.89 (0.73-1.08)  0.82 (0.66-1.02) |  |  |
|  |  |  |  | Q1: ≤0.5/0.1  Q2: >0.5/0.1-1.3/0.6  Q3: >1.3/0.6-2.4/1.2  Q4: >2.4/1.2-4.2/2.2  Q5: >4.2/2.2 | **(men)**  1.00 (ref)  0.88 (0.68-1.13)  0.87 (0.67-1.12)  0.85 (0.64-1.12)  0.78 (0.58-1.06) |  |  |
|  |  |  |  | Q1: ≤0.5/0.1  Q2: >0.5/0.1-1.3/0.6  Q3: >1.3/0.6-2.4/1.2  Q4: >2.4/1.2-4.2/2.2  Q5: >4.2/2.2 | **(women)**  1.00 (ref)  0.94 (0.73-1.22)  0.97 (0.74-1.28)  0.93 (0.70-1.23)  0.86 (0.63-1.17) |  |  |
| Valentina Rosato^5^,  2018,  Italy | Case-control study (hospital-based) | 688 (incidence) | 2204 men and women; 22y | Processed meat (g/d) |  | Sex, age, study  center, year of interview, education, tobacco smoking, alcohol drinking, body mass index, vegetables consumption, fruit consumption, and total energy intake | 6 |
|  |  |  |  | 10-20 | 1.31 (1.03-1.68) |  |  |
|  |  |  |  | >20 | 1.46 (1.15-1.85) |  |  |
| Jessica L. Petrick^6^,  2020,  United States | Cohort study | 168 (incidence) | 33151 African American women; 23y | Red and processed meat;  Quartile  (g/day) and frequency  (85g/week) |  | Age, BMI, smoking, alcohol, education, vigorous activity, diabetes, energy | 8 |
|  |  |  |  | Quartile  Q1: 9.0±4.8  Q2: 24.4±4.5  Q3: 42.8±6.7  Q4: 91.0±37.1 | **Total red meat**  1.00 (ref)  0.85 (0.55-1.31)  1.03 (0.67-1.60)  1.19 (0.73-1.92) |  |  |
|  |  |  |  | Frequency |  |  |  |
|  |  |  |  | <1/week  1-3/week  >3/week | 1.00 (ref)  0.65 (0.42-1.01)  0.97 (0.63-1.61) |  |  |
|  |  |  |  | Quartile  Q1: 7.1±4.3  Q2: 18.6±5.6  Q3: 32.3±9.0  Q4: 69.7±34.0 | **Unprocessed red meat**  1.00 (ref)  0.74 (0.48-1.14)  0.96 (0.63-1.46)  1.01 (0.63-1.61) |  |  |
|  |  |  |  | Frequency |  |  |  |
|  |  |  |  | <1/week  1-3/week  >3/week | 1.00 (ref)  0.83 (0.57-1.21)  0.99 (0.64-1.55) |  |  |
|  |  |  |  | Quartile  Q1: 2.1±2.5  Q2: 6.4±5.2  Q3: 11.9±8.6  Q4: 23.9±20.2 | **Processed meat**  1.00 (ref)  0.95 (0.63-1.44)  0.83 (0.54-1.28)  0.79 (0.49-1.25) |  |  |
|  |  |  |  | Frequency |  |  |  |
|  |  |  |  | <1/week  1-3/week  >3/week | 1.00 (ref)  0.99 (0.70-1.39)  0.59 (0.25-1.38) |  |  |
| Brian Z. Huang^7^,  2021,  United States | Cohort study (MEC) | 1618 (incidence and mortality) | 184542 men and women, 21y | Red meat;  Quartile  (g per 1000 kcal/d) |  | Age, sex, ethnicity, smoking, BMI, diabetes, family history, alcohol, caloris | 8 |
|  |  |  |  | Q1: 0.0-14.1  Q2: 14.1-23.9  Q3: 23.9-35.2  Q4: 35.2-216.5 | 1.00 (ref)  1.25 (1.08-1.43)  1.16 (1.00-1.34)  1.18 (1.02-1.37) |  |  |
| Brian Z. Huang^7^,  2021,  United States | Cohort study (SCCS) | 266 (incidence and mortality) | 66793 men and women, 14y | Red meat;  Quartile  (g per 1000 kcal/d) |  | Age, sex, ethnicity, smoking, BMI, diabetes, family history, alcohol, caloris | 8 |
|  |  |  |  | Q1: 0.0-26.3  Q2: 26.3-43.3  Q3: 43.3-64.5  Q4: 64.5-428.7 | 1.00 (ref)  0.97 (0.68-1.38)  1.20 (0.85-1.70)  1.31 (0.93-1.86) |  |  |
| Donghui Li^8^,  2019,  United States | Case-control study | 1321 (incidence) | 1061 men and women, 6y | Doneness |  | Age, race, sex education, diabetes, smoking, alcohol, first-degree relative with cancer, BMI at age of 30 | 8 |
|  |  |  |  | Hamburger or cheeseburger | 1.07 (0.77-1.48) |  |  |
|  |  |  |  | Steak | 1.07 (0.77-1.48) |  |  |
|  |  |  |  | Pork chops | 1.01 (0.80-1.27) |  |  |
|  |  |  |  | Bacon | 1.16 (0.93-1.46) |  |  |

**References**

**Taunk P, Hecht E, Stolzenberg-Solomon R. Are meat and heme iron intake associated with pancreatic cancer? Results from the NIH-AARP diet and health cohort. Int J Cancer. 2016 May 1;138(9):2172-89. doi: 10.1002/ijc.29964. Epub 2016 Jan 18. PMID: 26666579; PMCID: PMC4764390.**

**^2^ Beaney AJ, Banim PJR, Luben R, Lentjes MAH, Khaw KT, Hart AR. Higher Meat Intake Is Positively Associated With Higher Risk of Developing Pancreatic Cancer in an Age-Dependent Manner and Are Modified by Plasma Antioxidants: A Prospective Cohort Study (EPIC-Norfolk) Using Data From Food Diaries. Pancreas. 2017 May/Jun;46(5):672-678. doi: 10.1097/MPA.0000000000000819. PMID: 28375948; PMCID: PMC5400064.**

3 **Quist AJL, Inoue-Choi M, Weyer PJ, Anderson KE, Cantor KP, Krasner S, Freeman LEB, Ward MH, Jones RR. Ingested nitrate and nitrite, disinfection by-products, and pancreatic cancer risk in postmenopausal women. Int J Cancer. 2018 Jan 15;142(2):251-261. doi: 10.1002/ijc.31055. Epub 2017 Oct 25. PMID: 28921575; PMCID: PMC5788281.**

4 **McCullough ML, Jacobs EJ, Shah R, Campbell PT, Wang Y, Hartman TJ, Gapstur SM. Meat consumption and pancreatic cancer risk among men and women in the Cancer Prevention Study-II Nutrition Cohort. Cancer Causes Control. 2018 Jan;29(1):125-133. doi: 10.1007/s10552-017-0984-x. Epub 2017 Nov 28. PMID: 29185090.**

5 **Rosato V, Kawakita D, Negri E, Serraino D, Garavello W, Montella M, Decarli A, La Vecchia C, Ferraroni M. Processed meat and risk of selected digestive tract and laryngeal cancers. Eur J Clin Nutr. 2019 Jan;73(1):141-149. doi: 10.1038/s41430-018-0153-7. Epub 2018 Apr 16. PMID: 29662231.**

6 **Petrick JL, Castro-Webb N, Gerlovin H, Bethea TN, Li S, Ruiz-Narváez EA, Rosenberg L, Palmer JR. A Prospective Analysis of Intake of Red and Processed Meat in Relation to Pancreatic Cancer among African American Women. Cancer Epidemiol Biomarkers Prev. 2020 Sep;29(9):1775-1783. doi: 10.1158/1055-9965.EPI-20-0048. Epub 2020 Jul 1. PMID: 32611583; PMCID: PMC7484450.**

7 **Huang BZ, Wang S, Bogumil D, Wilkens LR, Wu L, Blot WJ, Zheng W, Shu XO, Pandol SJ, Le Marchand L, Setiawan VW. Red meat consumption, cooking mutagens, NAT1/2 genotypes and pancreatic cancer risk in two ethnically diverse prospective cohorts. Int J Cancer. 2021 Apr 12:10.1002/ijc.33598. doi: 10.1002/ijc.33598. Epub ahead of print. PMID: 33844845; PMCID: PMC8594451.**

8 **Li D, Tang H, Wei P, Zheng J, Daniel CR, Hassan MM. Vitamin C and Vitamin E Mitigate the Risk of Pancreatic Ductal Adenocarcinoma from Meat-Derived Mutagen Exposure in Adults in a Case-Control Study. J Nutr. 2019 Aug 1;149(8):1443-1450. doi: 10.1093/jn/nxz081. PMID: 31100111; PMCID: PMC6686056.**
